# Identification of Novel Hypoxia Response Genes in Human Glioma Cell Line A172 

**Published:** 2013-05

**Authors:** Fatemeh Baghbani, Reza Raoofian, Mohammad Hasanzadeh Nazarabadi, Tayebeh Hamzehloei, Mohammad Soukhtanloo, Mansur Heidari, Seyed Morteza Afsharzadeh, Sahar Shekouhi, Fahimeh Moradi, Abdol-Azim Sarli, Javad Zavar-Reza, Majid Mojarrad

**Affiliations:** 1 Department of Medical Genetics, School of Medicine, Mashhad University of Medical Sciences, Mashhad, Iran; 2 Department of Clinical Biochemistry, School of Medicine, Mashhad University of Medical Sciences, Mashhad, Iran; 3 Department of Medical Genetics, School of Medicine, Tehran University of Medical Sciences, Tehran, Iran; 4 Department of Clinical Biochemistry, School of Medicine, Shahid Sadoughi University of Medical Sciences, Yazd, Iran

**Keywords:** cDNA-AFLP, Glioblastoma, Hypoxia

## Abstract

***Objective(s):*** Hypoxia is a serious challenge for treatment of solid tumors. This condition has been manifested to exert significant therapeutic effects on glioblastoma multiform or (WHO) astrocytoma grade IV. Hypoxia contributes numerous changes in cellular mechanisms such as angiogenesis, metastasis and apoptosis evasion. Furthermore, in molecular level, hypoxia can cause induction of DNA breaks in tumor cells. Identification of mechanisms responsible for these effects can lead to designing more efficient therapeutic strategies against tumor progression which results in improvement of patient prognosis.

***Materials and Methods*** ***:*** In order to identify more hypoxia regulated genes which may have a role in glioblastoma progression, cDNA-AFLP was optimized as a Differential display method which is able to identify and isolate transcripts with no prior sequence knowledge.

***Results:*** Using this method, the current study identified 120 Transcription Derived Fragments (TDFs) which were completely differentially regulated in response to hypoxia. By sequence homology searching, the current study could detect 22 completely differentially regulated known genes and two unknown sequence matching with two chromosome contig and four sequence matches with some Expressed Sequence Tags (ESTs).

***Conclusion: ***Further characterizing of these genes may help to achieve better understanding of hypoxia mediated phenotype change in tumor cells.

## Introduction

Tumor hypoxia is a common feature of most solid tumors, and serves as a critical factor in tumor progression (1-5).

Several studies have shown that impaired balance of oxygen supplies, results from structurally and functionally irregular and disrupted diffusion conditions of tumor vascular system. These conditions along with unlimited fast proliferation of tumor cells are the main causes of hypoxic stress in tumor cells (6-8).

This stress condition exerts some adverse effects on neoplastic cells, in genome, transcriptome and metabolome homeostasis levels. These changes act as a natural selection force which affects tumor cells and leads to either tumor cells death or adaptation of these cells to hypoxia. Adapted cells represent a more aggressive behavior (4, 9-11). Hypoxia causes clonal expansion of tumor cells which are resistant to apoptosis, more invasive, metastatic and refractory to treatment (12-14).

As a clinical result, tumor hypoxia has direct correlation with poor prognosis in majority of solid tumors.

According to these findings, good understanding of molecular mechanisms of hypoxia mediated changes in tumor cells may help to find more effective therapeutic targets (8, 10, 12).

Glioblastoma multiform (GBM) or grade IV astrocytoma (based on World Health Organization classification) originating from glial tissue of brain, is the most common and aggressive form of intracranial primary brain tumors (8).

Fast growing, deep infiltration of tumor cells into surrounding healthy brain parenchyma, existence of hypoxic and necrotic regions and also poor prognosis are some inherent features of these lethal tumors (7, 8).

The median survival time for patients with glioblastoma is only 12–15 months, and the 3% 5-year survival rate is significantly lower than the 60% survival rate note for other brain tumors such as oligodendroglia and medulloblastoma (10).

Usual presence of excessive hypoxic and necrotic regions indicates that hypoxic stress plays a vital role in growth and invasiveness of tumor cells.

Since hypoxia is a potent controller of gene expression, characterization of hypoxia-regulated genes is a means to study the molecular response to hypoxic stress.

The current study aimed to elaborate the current knowledge of altered gene expression in high-grade astrocytoma and to identificate of additional oncogenes or tumor suppressor genes involved in evolution of glioblastoma multiform tumors.

 The present experiment used CDNA-AFLP method to assess A172 cell line gene expression profile in response to hypoxia condition.

## Materials and Methods

***Cell lines***

Human Glioma cell line A172 was obtained from National Cell Bank of Iran, Pasteur Institute of Iran (Tehran, Iran). 5×10^5^ cells were cultured in RPMI-1640 medium supplemented with 10% fetal bovine serum, 2 mM l-glutamine, 100 U/ml penicillin and 100 µg/ml streptomycin to 60% confluence. Cells were synchronized by overnight serum deprivation before hypoxia treatment.

***Hypoxia treatment***

After synchronization (as performed in triplicate), the medium were replaced by 10% FBS containing RPMI 1640 medium and cells were either placed overnight in an CO_2_/O_2_ incubator with 5% O_2_ and 5% CO_2_ at 37°C as hypoxic condition , and 21%O_2_, 5%CO_2_ at 37°C as normoxic condition.

***Total RNA isolation***

Total RNA from cells was extracted by using TriPure reagent (Roche) according to manufacturer’s instruction.

In summary, after removal of medium, 1 ml of TriPure reagent was added to each flask and cell lysates were homogenized by pipetting. RNA containing phase was isolated by adding 200 μl of chloroform and centrifugation. Total RNA was precipitated by adding 600 μl isopropanol and centrifugation. Finally RNA pellet was washed with 75% ethanol and after air drying; RNA was resolved in 40 μl of distilled water (DW).

***DNase treatment***

To eliminate genomic DNA contamination, RNA samples were treated by RNase free DNaseI (promega) according to manufactures instruction. Finally, integrity and concentration of RNA samples were evaluated by agarose gel electrophoresis and spectrophotometry with Nanodrop 2000.

***cDNA synthesis***

1 μg of RNA was applied to cDNA synthesis by MuMLV1 reverse transcriptase (Fermentas) according to manufacturer’s instruction.

20 μl of single strand cDNA was used to synthesize the second strand of DNA by using Klenow fragment of *E. Coli* DNA polymerase I. Reaction mixture was containing, 1X DNA Pol I buffer, 0.5 mM from each dNTP, 20 μl of CDNA and 80 unit of Klenow fragment. Finally, reaction mixture was adjusted to 150 μl by adding distilled water.

Reaction was performed by incubation at 16 C for 1 hr. After purification by ethanol precipitation, concentration of resulted dsDNA was evaluated by Nanodrop 2000.

***Enzymatic digestion and linker ligation***

dsDNA was partially digested by MboI and MseI enzymes to obtain a heterogeneous dsDNA mixture with 100-1000 bp in length. Reaction mixture was containing ~ 1 µg of dsDNA, 3 units of each restriction enzymes and 1x of reaction buffer. Reaction was performed in 37 C for 1hr.

Digested DNA samples were purified using silica gel and applied in ligation reaction by adding AFLP Adaptors to the end of DNA fragment. Reaction mixture was containing all of double digested DNA, 0.4 µM of each adaptor, 1X ligation buffer and 5 unit of T4 ligase. Reaction volume was adjusted to 20 µl by adding distilled water. Reaction was performed in 4°C overnight.

Ligation product was purified by ethanol precipitation and used as PCR reaction template.

***Preamplification and selective amplification***

Amplification of DNA fragment was performed in three steps by three sets of primers (Table 1). In each step DNA product of previous step was diluted with the ratio of 1:10 in DW (distilled water). Then, 1 μl of diluted DNA was applied as template in 25 μl reaction buffer containing 1x PCR buffer, 100 μM of each dNTP, 2 mM MgCl_2_, 0.2 μM of each primer and 1 unit of Taq Hot Start DNA polymerase.

PCR reaction was performed in 2720 ABI thermal cycler which is programmed for 30 cycles of denaturation at 94°C for 40 sec, 64°C for 40 sec and 72°C for 2 min; an additional step of denaturation at 95°C for 10min preceded the first cycle and another step of extension at 72°C for 7 min was extended at the end of reaction.

PCR products of hypoxia and normoxic samples from selective PCR reaction were subjected to electrophoresis on 10% polyacrylamide gel in parallel.

***Isolation, re-amplification and sequencing***

After silver staining of gels, transcription-derived fragments (TDFs) were compared in hypoxia and normoxic sample lanes. TDFs which were absent in one of the lanes, were scored as completely differentially expressed. Desired TDFs were excised from the gel and after DNA extraction, were subjected for re-amplification with a temperature profile as mentioned above.

Subsequently PCR products were electrophoresed through 2% agarose gel and then reamplifed products were extracted from agarose gel by DNA extraction from gel kit (bioneer) as recommended by the manufacturer. Finally, these fragments were sequenced and were analyzed by homology searching databases such as BLAST software.

## Results

To identify new induced genes expressed under the hypoxic stress, a cDNA-AFLP screen was carried out.

***General and selective amplification of transcriptome***

After partial enzymatic digestion of dsDNA and ligation of adaptors into the end of DNA fragments, PCR amplification was done by three rounds of PCR. 

**Figure 1:**
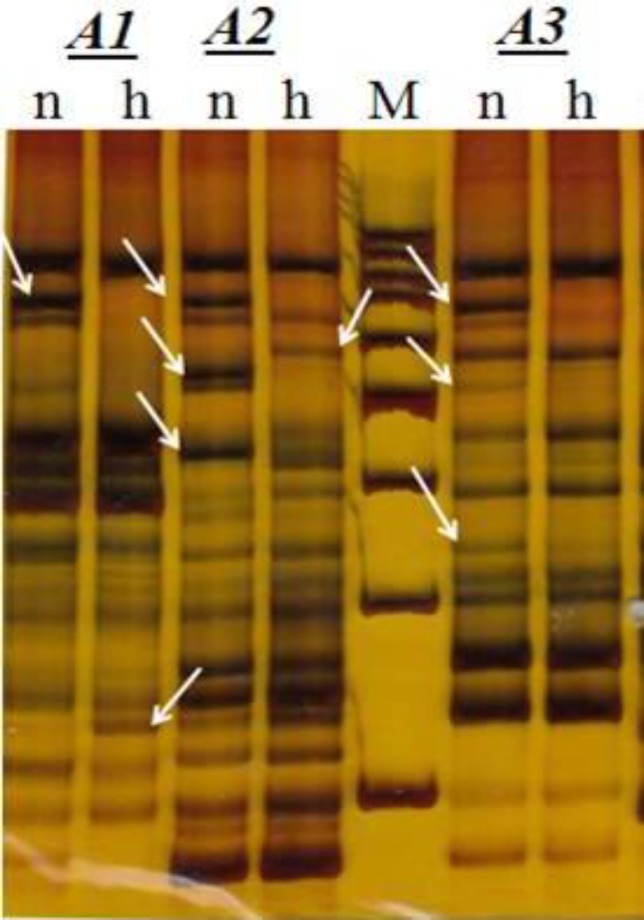
Expression of Hypoxia and Normoxia transcripts displayed by cDNA-AFLP. An example showing selective amplification with three diffrent primer combination (Ms5 as forward primer and Mb 5-7 as reverse primers in A1-A3 respectively) of A172 Glioma cell line exposed to Hypoxia stress. Arrows indicate transcript-derived fragments that show differential expression in either in hypoxia (h) or normoxia condition (n). M represents 100bp molecular weight marker.

Final PCR products were separated by polyacrylamide gel electrophoresis and visualized by silver nitrate staining (Figure 1). After evaluation of approximately 5000 TDFs resulting from different primer combinations, about 120 bands were scored as completely differentially expressed. Seventy three out of 120 TDFs were hypoxia depressed and the remaining 47 TDFs were hypoxia induced. Desired TDFs undergo to DNA extraction and reamplification respectively.

By DNA sequence analysis of differentially expressed TDFs 22 known genes could be detected (Table 2). Furthermore, two TDFs were detected which matched with chromosome contig on chromosome 9 and 10. In addition, four TDFs were matched to Expressed Sequence Tags (ESTs).

**Table 1:** Sequence of Adaptors and PCR Primers used in cDNA-AFLP analysis

	Forward primer		Reverse primer	
	Name	Sequence	Name	Sequence
Adaptor sequences	Adap MseI	GTAGCGCGACGGCCAGTCGCGT	Adap MboI	GTAGCGCGACGGCCAGTCGCG
	Adap MseI	CATCGCGCTGCCGGTCAGCGCAAT	Adap MboI	CATCGCGCTGCCGGTCAGCGCCTAG
Preamplification primers	PreAmp primer	CATCGCGCTGCCGG		
First selective PCR primers	Ms1	gctGCCGGTCAGCGCAATg	Mb1	TGCCGGTCAGCGCCTAGg
	Ms2	gctGCCGGTCAGCGCAATc	Mb2	TGCCGGTCAGCGCCTAGc
	Ms3	cgctGCCGGTCAGCGCAATa	Mb3	cTGCCGGTCAGCGCCTAGa
	Ms4	cgctGCCGGTCAGCGCAATt	Mb4	cTGCCGGTCAGCGCCTAGt
Second selective PCR primers	Ms5	ctGCCGGTCAGCGCAATgg	Mb5	TGCCGGTCAGCGCCTAGga
	Ms6	ctGCCGGTCAGCGCAATgc	Mb6	TGCCGGTCAGCGCCTAGgt
	Ms7	gctGCCGGTCAGCGCAATga	Mb7	TGCCGGTCAGCGCCTAGgc
	Ms8	gctGCCGGTCAGCGCAATgt	Mb8	TGCCGGTCAGCGCCTAGgg
	Ms9	ctGCCGGTCAGCGCAATcg	Mb9	gCCGGTCAGCGCCTAGca
	Ms10	ctGCCGGTCAGCGCAATcc	Mb10	tgCCGGTCAGCGCCTAGct
	Ms11	gctGCCGGTCAGCGCAATca	Mb11	tgCCGGTCAGCGCCTAGcc
	Ms12	gctGCCGGTCAGCGCAATct	Mb12	gCCGGTCAGCGCCTAGcg
	Ms13	gctGCCGGTCAGCGCAATag	Mb13	ctgCCGGTCAGCGCCTAGaa
	Ms14	gctGCCGGTCAGCGCAATac	Mb14	ctgCCGGTCAGCGCCTAGat
	Ms15	cgctGCCGGTCAGCGCAATaa	Mb15	ctgCCGGTCAGCGCCTAGac
	Ms16	cgctGCCGGTCAGCGCAATat	Mb16	ctgCCGGTCAGCGCCTAGag
	Ms17	gctGCCGGTCAGCGCAATtg	Mb17	ctgCCGGTCAGCGCCTAGta
	Ms18	gctGCCGGTCAGCGCAATtc	Mb18	ctgCCGGTCAGCGCCTAGtt

**Table 2:** Summary of cDNA-AFLP analysis results

Official symbol	Location	Official full name	Function
DHX8	17q21.31	DEAH (Asp-Glu-Ala-His) box polypeptide 8	nucleotide, RNA splicing
ODF2L	9q34.11	outer dense fiber of sperm tails 2-like	maintain the passive elastic structures and elastic recoil of the sperm
TOR1AIP2 (LULL1)	1q25.2	torsin A interacting protein 2	chaperon like function
CTSC	11q13.1	a lysosomal protease	cysteine proteinase, involved in degradative processes during tumor progression
HSP90AB2P	4p15.33	heat shock protein 90kDa alpha (cytosolic), class B member 2 (pseudogene)	pseudogene
UBAP1	9p13.3	Ubiquitin associated protein 1	ubiquitination pathway
ENO2	12p13.31	During neurons development	neuro protective
DDHD1	14q21	DDHD domain containing 1	cellular trafficking transport
CCDC144B	17p.11.2	coiled-coil domain containing 144B	pseudogene
RPL27A	11p15	ribosomal protein L27a	60S ribosomal protein L27A
TMEM212	3q26.31	transmembrane protein 212	unknown
XRCC2	7q36.1	X-ray repair cross-complementing group 2	homologous recombination repair (HRR) pathway
SAT1	Xp22.1	spermidine/spermine-N acetyltransferase-1	Polyamines homeostasis
SMS	Xp22.11	homo sapiens spermine synthase (SMS), mRNA	brain development and cognitive function
XIAP	Xq25	X-linked inhibitor of apoptosis (XIAP)-associated factor 1	X-linked inhibitor of apoptosis
ZNF197	3p21	zinc finger protein 197	transcription factor
EIF3K	19q13.2	translation initiation factor 3	translation initiation factor 3
MTRNR2L1	17p11.2	Humanin(MTRNR2L1)	cell life, antiapoptosis
CFLAR	2q33.1	CASP8 and FADD-like apoptosis regulator	Apoptosis regulator
PABPC1	8q22.3	poly(A) binding protein, cytoplasmic 1-like	mRNA translation and stability
TOR1A	9q34.11	torsin family 1, member A (torsin A)	synaptic functioning
RPLP0	12q24.2	ribosomal protein, large, P0	protein, translation/synthesis
POP4	19q13.11	processing of precursor 4, ribonuclease P/MRP subunit	involved in tRNA processing
			

## Discussion

For a long time, identification of molecular pathways involved in progression and development of solid tumor were the main concern of scientists for cancer therapy. During the past decade, numerous investigations were carried out by focus on amount of oxygenation of solid tumors which result to considerable information about oxygen homeostasis in tumors. Some of these remarkable findings are: lower O_2_ pressure of tumor tissue compared to origin, existence of hypoxic regions in tumors regardless of tumor features such as clinical size, grade, histology and position of tumor, as well as severe hypoxic stress in recurrent tumors compared to primary tumors (3).

An accumulating number of evidence about hypoxia-induced changes from the genomic to the posttranslational levels provide clearer insight into crucial role of oxygen homeostasis in cancer promotion, however, more studies are needed to precisely decode hypoxia mediated phenotype changes (15).

For better understanding of glioma cell transcriptome alterations in response to hypoxia, cDNA-AFLP was employed. By 256 primer combinations, about 5000 TDFs could be screened.

Of these bands, 120 bands were scored as completely differentially regulated. By sequencing and homology searching of these bands, 22 genes were known as hypoxia regulated.

These genes can be divided into some functional categories which will be discussed here.

***Genes regulating polyamine homeostasis***

Two of the genes induced by hypoxia were involved in polyamine homeostasis. 

Spermine/Spermidine Acetyl Transferase 1 (SAT1) is a key regulator of polyamine homeostasis, which is essential in central cellular processes including proliferation and differentiation (16).

A large number of documents indicate that SAT-1 activity is under precise regulation of several microenvironment factors such as toxic agents, hormones, growth factors and also polyamines (16). Furthermore, recent evidences have shown that cell oxygen pressure directly affects enzyme activity.

According to recent studies, SAT-1 has a contradictory role in neoplastic promotion which is relevant to tumor tissue. Some investigations have shown that high level expression of SAT1, display tumor-suppressive effects while other studies point to neoplastic initiator role of SAT1 overexpression (16, 17).

The second polyamine regulating enzyme which was detected as hypoxia induced by the current experiment is spermine synthase (SMS). This enzyme catalyzes the production of the polyamine spermine from the shorter chain of polyamine spermidine (18). SMS is widely expressed in brain and is responsible for brain development and cognitive function (19). Spermine has neuroprotective effects when brain damage happens (20). Recently Nikhil *et- al* reports that SMS gene is one of the 55 survival genes that their suppression leads to glioma cell death (21).

***Genes which are important in gene expression and protein synthesis regulation***

Maintenance of cell homeostasis and regulation of cell proliferation importantly depend on regulating the process of gene expression and protein synthesis.

Expression of five genes which involved in this process was detected.

Eukaryotic translation initiation factor 3 (*EIF3*) genes induced in hypoxia condition. EIF3 is a member of multi subunit complex containing at least 12 subunits which is required for several steps of protein synthesis initiation (22).

Although there are a few evidences of *EIF3* complex expression roles in cancer development (23), in recent investigations, *EIF3* overexpression has been observed in breast and prostate cancer (24); also high level expression of *EIF3-p110 *subunit has been seen in testicular seminoma (24, 25).

*PABPC1* was another newly identified gene as a hypoxia regulated gene. It encodes a protein with influence on mRNA stability and transfer between the nucleus and cytoplasm (26). This protein directly binds to the poly (A) tail of polyadenylated and intron containing pre-mRNAs, and effects on translation and stability of mRNA (27). According to recent studies, *PABPC1 depletion* of cells by siRNA results to enhance serine 46 phosphorylation of p53, and eventually trigger Bax apoptosis pathway (28). Stability of c-fos mRNA, which encodes a transcription factor, is also modulated by *PABPC1* (26).

Furthermore, *PABPC1 *involves in processing of primary microRNAs (29). It seems that hypoxic cells escape from apoptosis by expressing *PABPC1 *and also change their microRNome toward becoming more resistant to environmental stresses.

ATP-dependent RNA helicase DHX8 is a crucial member of the eukaryotic pre-mRNA splicing machinery and belongs to DEAD box proteins with conserved motif Asp-Glu-Ala-Asp (DEAD) which are members of helicase super family II. DHX8 encodes a DEAD box protein which is homolog to yeast Prp22. This protein facilitates nuclear transport of mature mRNA from the U5 snRNP of the spliceosome complex by releasing the RNA. It may have a general regulatory role in cell transcription (30, 31).

One of the most important gene expression regulatory gene families in eukaryotic cell is the zinc finger containing genes. According to the obtained results, ZNF197 gene is a hypoxia regulated gene in glioma cells. *ZNF197* is located in a cluster of zinc finger genes at chromosome 3p21 (32). This gene is highly expressed in esophagus, thyroid and reproductive system.* ZNF197* is also overexpressed in some thyroid papillary carcinomas. The role of this gene in mechanisms of hypoxia induced response is unknown (32-34).

Torsin A interacting protein 2a or LULL1 is another hypoxia induced gene detected in the current study. Because of sequence similarity between LULL1 protein and chaperons in AAA-domain, it seems that Torsin A interacting protein has chaperon like function in ER lumen and by conformational alteration of substrate such as Torsin A, plays an important role in brain cells (35). Also dysfunction of this transmembrane protein results in an autosomal dominant childhood-onset neurological disease DYT1 Dystonia (35, 36).

***Genes which protect cells from apoptosis***

As mentioned before, one of the most important effects of hypoxia on tumor cells is expansion of cells which are resistant to apoptosis. The current study found two antiapoptotic genes which are overexpressed in hypoxia condition.

MTRNR2L1 or Humanin (HN) is a 24-amino acid peptide with anti-apoptotic features, which was firstly identified as a neuroprotective factor against Alzheimer's disease (AD) (37).

Several recent *in vitro* studies have confirmed that the HN peptide protects neurons from apoptosis by mechanisms such as inhibition of OGD-induced neuronal apoptosis, ASK/JNK mediated neuronal cell death, and mitochondrial related Bax apoptosis (38).

More recently, it has been demonstrated that HN involves in physiological mechanisms which are promoting cell survival in stressful conditions, such as neurodegeneration, inflammation or energy crisis (39, 40).

Additionally, it has been reported that Humanin increases ATP biosynthesis in human rhabdomyosarcoma TE671 cells cultured under serum-free conditions (41). So, this protein may be involved in mitochondrial related diseases or brain ischemia.

Humanin, also through the STAT3 dependent antiapoptotic signal transduction cascade has correlation with oncogenesis (42).

X-linked inhibitor of apoptosis protein (XIAP) is another anti apoptotic gene which is induced by hypoxia in the current experiment. XIAP belongs to inhibitor of apoptosis proteins family of caspase inhibitors. According to recent studies, it seems that overexpression of XIAP protein has apoptosis inhibitory effect on both the initiation and execution phases of the caspase cascade and finally leads to suppression of the programmed cell death (43). Since *XIAP *overexpression is directly related to many diseases such as cancer progression, autoimmune and neurodegenerative disease, it is an attractive target for novel therapeutic agents for the treatment of malignancy as a new way to counteract cancer and overcome drug resistance and poor clinical outcome (44).

XIAP, by formation of a complex with the TAK1 kinase and TAB1, can result in activation of apoptotic signaling pathways including NF-kB, JNK, MAP kinase, and the ubiquitin proteosome pathways (45). Furthermore, XIAP plays a crucial role in a variety of cellular functions including immune regulation, cell division and differentiation, cell migration, morphogenesis, and heavy metal metabolism (44).

Especially it has been reported that *XIAP* is overexpressed in glioblastoma (46), and it is supposed to be associated with drug resistance and poor prognosis of these patients.

 The current study also identified *CFLAR* induction by hypoxia. CFLAR (CASP8 and FADD-like apoptosis regulator) gene located on 2q33.1 which encodes a regulatory protein of apoptosis pathway and plays a significant role in the regulation of apoptosis and is structurally similar to caspase-8 which blocks death receptor-mediated apoptosis by inhibiting caspase 8 (47).

***Genes involved in DNA repair***

One of the well-known effects of hypoxia stress is loss of genomic integrity and DNA breakage. So, hypoxic cells adapt with this environmental pressure by over activating of DNA repair systems.

X-ray repair cross-complementing group-2 (*XRCC2*) gene is an important member of mammalian RecA/Rad51 family proteins which are related to the homologous recombination repair (HRR) pathway required for correct chromosome segregation and the repair of DSBs (48). Because of its interaction with some tumor suppressor genes such as *brca1* and *brca2*, *c-abl*, *p53*, it seems that *XRCC2* is involved in tumor progression (48, 49). 

Several investigations point to the role of *XRCC2 mutations* and polymorphisms in various cancers such as breast cancer, colorectal cancer, brain cancer, oral and Lynch syndrome and esophageal adenocarcinoma (50).

Overexpression of X-ray repair cross-complementing group 2(XRCC2) is a hallmark of neoplastic cells and especially in glioblastoma tumor cells leads to resistance against anticancer drugs Temozolomide (TMZ) (51).

***Cancer/testis specific gene***

*ODF2* cancer/testis gene was found as a *de novo* transcriptional target of hypoxia. *ODF2 *is one of the major components of the cytoskeleton of the sperm tail. 

*ODF2 *by interaction with other proteins, like Plk1, plays a crucial role in spindle formation and the progression into mitosis (52).

*ODF2 has* a testis restricted expression pattern and does not express in normal tissues. Ectopic expression of this gene has been detected in prostate cancer and basal cell carcinoma. 

In addition to these results, the current study detected hypoxic induced expression of 4 EST and also 2 chromosome contig sequences, which indicate the presence of other unknown genes in hypoxic response of cells.

cDNA-AFLP which was first developed by Vos *et- al* (1995) is a powerful approach that facilitates identification and isolation of transcripts (53). Also, it can discover any variation between two global expression patterns without pervious knowledge of sequence. In addition, it is able to amplify any infrequent transcripts from transcriptome pool.

## Conclusion

The present study found signiﬁcant variations in gene expression among hypoxia and normoxic cultured glioma cells. Some of these results are reported for the first time and when further characterized may give us precise insights to the process of transcriptome re-modeling by hypoxia.
